# A lipid nanoparticle encapsulated CPA-CTD mRNA vaccine provides protection against *Clostridium perfringens*-driven diseases

**DOI:** 10.3389/fimmu.2025.1748171

**Published:** 2026-01-08

**Authors:** Qi Jia, Haoyu Xiang, Ting Le, Jing Wang, Jitao Chang, Fang Wang, Chao Sun, Wanbo Tai, Zhigang Jiang, Xin Yin

**Affiliations:** 1 State Key Laboratory of Animal Disease Control and Prevention, Harbin Veterinary Research Institute, Chinese Academy of Agricultural Sciences, Harbin, China; 2 College of Animal Science and Technology, Ningxia University, Yinchuan, China; 3 TERRA Teaching and Research Center, Gembloux Agro-Bio Tech, University of Liège, Gembloux, Belgium; 4 Institute of Infectious Diseases, Shenzhen Bay Laboratory, Shenzhen, China

**Keywords:** *Clostridium perfringens* alpha-toxin, enterotoxemia, gas gangrene, mRNA vaccine, protective efficacy

## Abstract

**Introduction:**

*Clostridium perfringens* (*C. perfringens*), a ubiquitous Gram-positive bacterium in the environment and mammalian gut flora, is a leading cause of enterotoxemia in animals, necrotizing enteritis in humans and animals, and gas gangrene in both, attributed to its diverse exotoxin profile. Alpha-toxin, a pivotal virulence factor produced by all *C. perfringens* serotypes, plays a central role in the pathogenicity of these diseases.

**Methods:**

Here, we engineered a lipid nanoparticle encapsulated CPA-CTD mRNA vaccine targeting the conserved C-terminal domain of *C. perfringens* alpha-toxin and rigorously assessed its immunogenicity and protective efficacy in mouse and bovine models.

**Results:**

The CPA-CTD mRNA vaccine induced strong humoral and cellular immune responses in mice, particularly in promoting the rapid production of specific IgG and mucosal IgA antibodies, as well as enhancing T cell immune responses, surpassing conventional subunit vaccines. Protection was confirmed in dual challenge models --enterotoxemia and gas gangrene --where the vaccine provided complete immunity against lethal doses of alpha-toxin and *C. perfringens* infection. In cattle, the CPA-CTD mRNA vaccine induced high-titer IgG antibodies and toxin-neutralizing antibodies. Notably, immunization of pregnant cows led to efficient transfer of these antibodies via colostrum to newborn calves, providing passive protection.

**Discussion:**

These results demonstrate that the CPA-CTD mRNA vaccine provides rapid and robust immune protection against *C. perfringens* alpha-toxin-associated diseases, with promising potential for applications in both veterinary and human health.

## 1 Introduction

**Figure 1:**
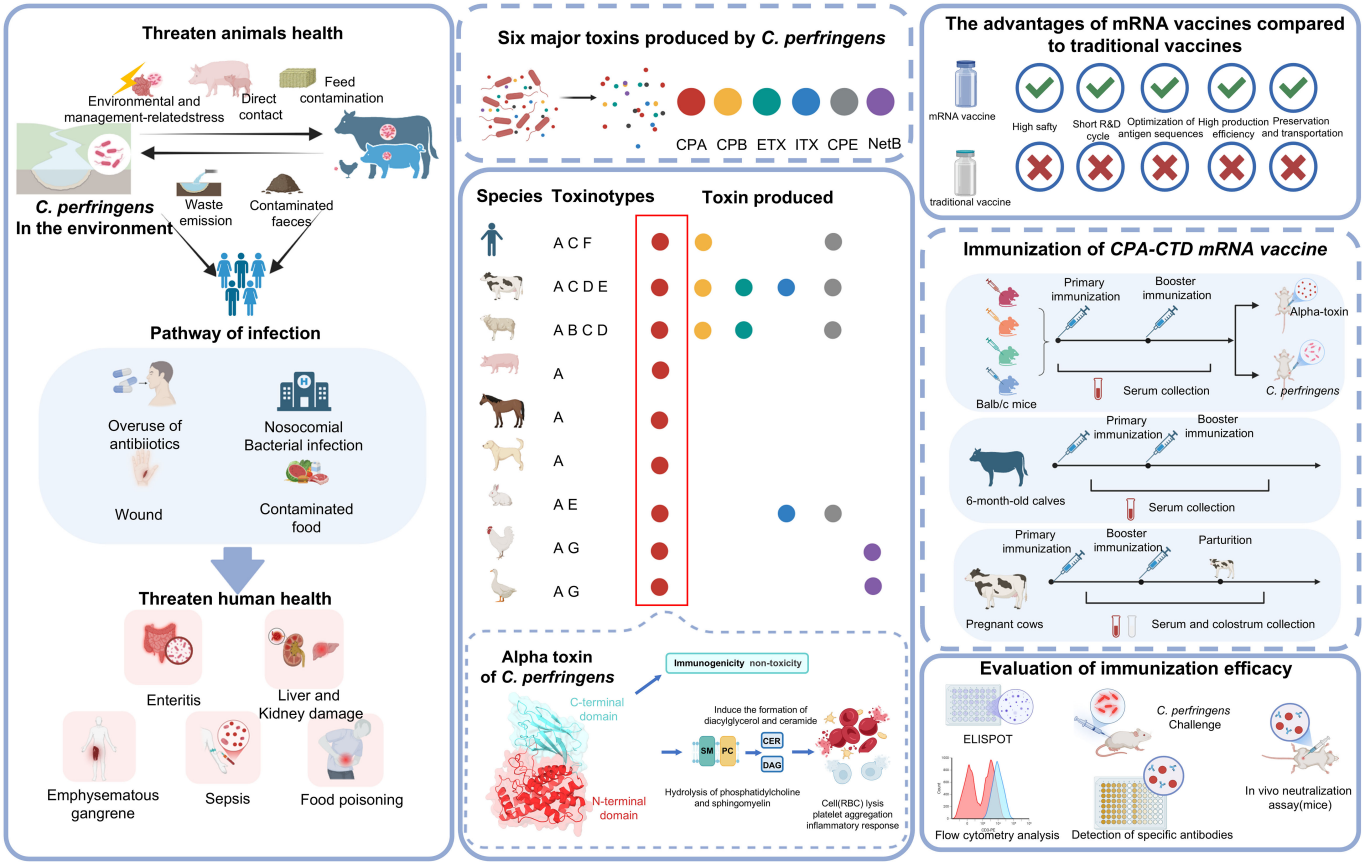
The impact of *C. perfringens* on humans and animals, the pivotal role of alpha-toxin in its pathogenicity, and the development of mRNA vaccines targeting alpha-toxin.

*Clostridium perfringens* (*C. perfringens*) is a Gram-positive, spore-forming, rod-shaped bacterium known for its rapid proliferation and unique anaerobic metabolism. Ubiquitous in soil, water, and the gastrointestinal tracts of humans and animals, this pathogen exploits diverse ecological niches to drive widespread contamination, causing diseases such as enterotoxemia in animals (1), necrotizing enteritis and gas gangrene in humans and animals (2–6), and food poisoning in humans, thereby posing a persistent threat to public health through severe infections and inflicting substantial economic losses in livestock industries due to high morbidity and mortality (7–11) (**Figure 1**). The pathogenicity of bacterium is driven by an extensive repertoire of over 20 exotoxins and enzymes, which vary across strains. *C. perfringens* is classified into seven toxinotypes (A–G) based on the production of six major exotoxins: alpha, beta, epsilon, iota, enterotoxin, and NetB (11, 12) (**Figure 1**).

Alpha-toxin, a zinc-dependent phospholipase C enzyme universally expressed by all *C. perfringens* toxinotypes, is a cornerstone of its virulence, mediating extensive tissue damage and immune dysregulation. This toxin hydrolyzes phospholipids in host cell membranes, disrupting integrity and triggering hemolysis, necrosis, and inflammation, which amplify its pathogenic impact. The pivotal role of alpha-toxin is evident in a spectrum of severe diseases, including enterotoxemia in animals (13). necrotizing enteritis in humans and animals, and gas gangrene in both, often acting synergistically with other toxins to exacerbate clinical outcomes (14, 15) (**Figure 1**). Its consistent production across all strains and its ability to hydrolyze diverse phospholipids highlight its status as a primary etiological factor, making it a prime target for therapeutic and prophylactic strategies. Understanding molecular mechanisms of alpha-toxin—such as its lectin-like C-terminal domain’s role in membrane binding—offers critical insights into mitigating *C. perfringens*-associated pathology (16, 17) (**Figure 1**). Research targeting alpha-toxin is thus essential not only for unraveling its contribution to disease progression but also for developing innovative interventions, such as vaccines, to combat the devastating effects of this multifaceted pathogen on veterinary and human health worldwide.

Prevention of diseases associated with *C. perfringens* has traditionally depended on inactivated monovalent vaccines, multivalent formulations, or toxoid-based immunogens. These conventional methods trigger protective antibody responses in animals but face limitations such as inconsistent efficacy, poor stability, and safety issues, including pyrogenic reactions and allergic responses, often worsened by adjuvants and residual contaminants. Beyond these, subunit vaccines targeting toxins of *C. perfringens*, particularly alpha-toxin, have undergone exploration. Notably, the C-terminal domain of alpha-toxin demonstrates immunoprotective potential, capable of inducing neutralizing antibodies against pathology mediated by alpha-toxin in preclinical models (7, 18–20) (**Figure 1**). Given the ubiquitous expression of alpha-toxin and the pivotal role it plays in driving severe diseases, development of an effective vaccine targeting alpha-toxin remains critical to reduce the global burden of infections caused by *C. perfringens* (4, 20–23). However, subunit vaccines encounter significant drawbacks: production involves complex processes requiring extensive purification, dependence on adjuvants to enhance immunogenicity, and the need for multiple doses to achieve adequate protection limit their practicality and scalability. Consequently, a safe, stable, and efficient vaccine remains urgently needed. The advent of messenger RNA (mRNA) vaccines, delivered via the lipid nanoparticle (LNP), has transformed vaccine technology, yielding promising results against various viral pathogens (24–26), such as SARS-CoV-2, and a few bacterial pathogens (27). Rapid production, enhanced immunogenicity, and antigenic flexibility make mRNA platforms a compelling alternative for addressing *C. perfringens*.

Building on the potential of mRNA platforms to overcome limitations of conventional and subunit vaccines (28, 29), this study develops an mRNA vaccine utilizing a non-toxic sequence targeting the C-terminal domain (amino acids 247–370) of *C. perfringens* alpha-toxin (CPA-CTD), encapsulated in LNP to enhance delivery and immunogenicity (**Figure 1**). In mice, we test varying doses of CPA-CTD mRNA-LNP for CPA-specific humoral and cellular immunity, efficacy against subunit vaccines, and protection in enterotoxemia and gas gangrene challenge models (**Figure 1**). Moreover, evaluations in host cattle confirms the vaccine’s robust capacity to elicit active immunity through antibody production and passive immunity via colostrum transfer to calves (**Figure 1**). This study applies mRNA technology to the relatively underexplored area of bacterial toxins, specifically targeting *C. perfringens*. By targeting alpha-toxin, a key virulence factor, the vaccine addresses the urgent need for effective prevention and holds promise for reducing economic losses in livestock and health risks in human populations.

## 2 Materials and methods

### 2.1 Design and production of the CPA-CTD mRNA vaccine

The CPA-CTD mRNA vaccine was designed based on the C-terminal domain (amino acids 247–370) of the *C. perfringens* type A strain HLJ-A5 (Genbank: PQ858782), selected as the target sequence due to its non-toxic yet immunogenic properties. A mammalian codon-optimized DNA template encoding the CPA-CTD fragment was generated by fusing the coding sequence to a tPA signal peptide and adding a 100-nucleotide poly(A) tail. The DNA template was synthesized by Suzhou Ribo Life Science Co., Ltd. After linearization, *in vitro* transcription (IVT) was performed using the T7 High Yield RNA Transcription Kit (Vazyme), with N1-methylpseudouridine triphosphate included in the reaction to enhance mRNA stability and reduce innate immunogenicity. The IVT products were purified and subsequently capped using a Cap 1 capping system to support efficient translation initiation. The resulting mRNA was encapsulated into LNP comprising cationic lipid SM-102, distearoylphosphatidylcholine (DSPC), cholesterol, and DMG-2000 (Sino-Bonag Corporation, China). Lipids were dissolved in anhydrous ethanol and mixed with the mRNA in 50 mM citrate buffer (pH 4.0) at a 1:3 volumetric ratio using microfluidic technology. The LNP formulation was then diluted with phosphate-buffered saline (PBS) and concentrated via ultracentrifugation (Amicon Ultra-15, Millipore, USA) to yield the CPA-CTD mRNA-LNP vaccine. The particle size and polydispersity index (PDI) of the resulting nanoparticles were then determined using a Zetasizer Nano (Malvern Panalytical, UK) to ensure their uniformity and stability.

### 2.2 Evaluation of CPA-CTD mRNA-LNP expression and cytotoxicity in multiple cells

HEK293T, BHK-21, and Vero cells were cultured in Dulbecco’s Modified Eagle Medium (DMEM; Gibco, USA) supplemented with 10% fetal bovine serum (FBS; Sigma, USA) and 1% penicillin-streptomycin (Gibco, USA) at 37 °C in a humidified 5% CO_2_ incubator. For transfection, cells were seeded into 24-well plates at a density of 2 × 10^5^ cells per well and incubated for 18 hours to adhere. CPA-CTD mRNA-LNP were then added at 1 μg per well in serum-free DMEM and incubated for 6 hours. The medium was replaced with complete DMEM, and cells were cultured for an additional 18 hours. A negative control group treated with an equivalent dose of empty LNP (lacking encapsulated mRNA) was included. CPA-CTD protein expression was confirmed using indirect immunofluorescence assay (IFA) and Western blot. Transfected cells were fixed with 4% paraformaldehyde for 15 minutes, permeabilized with 0.1% Triton X-100 for 15 minutes, and blocked with 5% bovine serum albumin (BSA) for 1 hour. A mouse CPA-CTD-specific monoclonal antibody at a 1:500 dilution (preserved in our laboratory) was applied and incubated overnight at 4°C, followed by incubation with Alexa-Fluor-488-conjugated goat anti-mouse IgG (Invitrogen, USA) for 1 hour at 37°C. Nuclei were stained with 0.01% DAPI, and images were captured using a fluorescence microscope (Leica, Germany). Western blot analysis involved lysing cells in Pierce IP lysis buffer (Thermo Scientific, USA) with protease inhibitors, separating proteins, transferring them to PVDF membranes, and probing with the CPA-CTD-specific monoclonal antibody at a 1:1000 dilution and Goat Anti-Mouse IgG (Fc specific)–HRP (Sigma, USA) for 1 hour at 37°C. Bands were visualized using a Touch Imager (eBLOT, China). Cytotoxicity was assessed by seeding cells into 96-well plates at a density of 1 × 10^5^ cells per well, transfecting with 0.5 μg CPA-CTD mRNA-LNP, and measuring viability after 48 hours with a CCK-8 assay (APExBIO, USA) at 450 nm, relative to untreated controls.

### 2.3 Immunization of animals with CPA-CTD mRNA vaccine

A total of 135 Specific pathogen-free (SPF) female BALB/c mice, aged 7 weeks, were obtained from Liaoning Changsheng Biotechnology Co., Ltd. (Liaoning, China). The mice were randomly divided into four groups and immunized intramuscularly (IM) twice, with a booster dose administered 21 days after the initial immunization at the same dosage. The CPA-CTD mRNA vaccine groups received doses of 20 μg or 5 μg, with 40 mice in each group. The subunit vaccine control group(n=30) was immunized with 20 μg of purified recombinant CPA-CTD protein (rCPA-CTD) mixed with ISA 15A VG adjuvant (Seppic, Paris, France) at an antigen-to-adjuvant ratio of 85:15. The blank control group (n=25) received an equivalent volume of phosphate-buffered saline (PBS). Blood and fecal samples were collected weekly following the initial immunization to monitor immune responses.

Fifteen 6-month-old calves with alpha-toxin neutralizing antibody titers below 1 were divided into three groups. Two groups, each consisting of 6 calves, received either 100 μg or 400 μg of the CPA-CTD mRNA vaccine, while the remaining 3 calves were administered 300 μL of PBS as controls. All calves underwent two intramuscular immunizations, 3 weeks apart. Rectal temperatures were measured daily at 8–9 a.m. and 4–5 p.m. for 14 days following each immunization, along with observations of mental state, appetite, and injection sites. Blood samples were collected weekly post-immunization to monitor antibody responses.

Six pregnant cows with alpha-toxin-neutralizing antibody titers below 1 were divided into two groups, with 3 cows in each group. One group received 400 μg of CPA-CTD mRNA vaccine, while the other was administered 300 μL of PBS as a control. Both groups underwent two intramuscular immunizations, 3 weeks apart. Serum samples from immunized cows, colostrum collected within 7 days post-calving, and serum from newborn calves were obtained. Subsequently, blood samples from the calves were collected weekly. All samples were analyzed to assess specific immune responses.

### 2.4 Enzyme-linked immunosorbent assay

ELISAs were conducted to measure IgG levels in serum and IgA levels in fecal samples. Ninety-six-well plates were coated overnight at 4°C with 200 ng of rCPA-CTD per well in suspended in carbonate buffer solution (100 mM, pH 9.6). Serum samples were heat-inactivated at 56°C for 30 minutes prior to testing. Fecal samples were processed by suspending 1 g in 1 mL of sterile PBS, centrifuging at 10,000 × g for 10 minutes at 4°C, and collecting the supernatant. Processed serum and fecal supernatants were serially diluted and added to the blocked plates. After incubation at 37°C for 1 hour, Goat Anti-Mouse IgG (Fc specific)–HRP (diluted at 1:10,000) or Rabbit Anti-Bovine IgG (whole molecule)−HRP (diluted at 1:10,000) was added to serum samples, while HRP-conjugated Goat Anti-Mouse IgA (diluted at 1:1,000; Proteintech, USA) was applied to fecal samples, followed by incubation at 37°C for 1 hour. TMB substrate solution (Solarbio, China) was then added and incubated for 10 minutes at room temperature. The reaction was stopped with 50 μL of 2 M H_2_SO_4_, and absorbance was measured at 450 nm using a Synergy H1 hybrid multimode microplate reader (BioTek, USA).

### 2.5 Lymphocyte proliferation assay

Splenic lymphocytes were isolated from immunized mice at 42 days post-vaccination (dpv) for the lymphocyte proliferation assay. Cell suspensions were prepared at a concentration of 5 × 10^6^ cells/mL, and 100 μL of the suspension was dispensed into each well of a 96-well plate. Lymphocytes were stimulated with 10 μg/mL of rCPA-CTD, with five replicate wells per sample. The plate was incubated at 37°C in a 5% CO_2_ incubator for 72 hours. Subsequently, 10 μL of CCK-8 solution (APExBIO, USA) was added to each well, followed by incubation at 37°C for 3 hours. Absorbance was measured at 450 nm, and the relative stimulation index was calculated as the ratio of the average absorbance of antigen-stimulated wells to unstimulated wells.

### 2.6 Flow cytometry

Splenic lymphocytes isolated from immunized mice at 35 dpv were transferred into 1.5 mL centrifuge tubes with 1 × 10^6^ cells per tube. Cells were washed once with PBS and resuspended in 300 μL of flow cytometry staining buffer (eBioscience, USA). Fluorescent antibodies—FITC-conjugated hamster anti-mouse CD3ϵ, PE-conjugated anti-mouse CD4, and APC-conjugated anti-mouse CD8a (BioLegend, USA)—were added, and the mixture was incubated at room temperature in the dark for 30 minutes. After centrifugation at 1,500 rpm for 5 minutes, the supernatant was discarded, and cells were washed twice with PBS. The cell pellet was resuspended in 500 μL of fluorescence preservation solution (0.15 M PBS, pH 7.4, with 2% glucose, 1% formaldehyde, and 0.1% NaN^3^). Flow cytometry analysis was performed using a Cytomics FC 500 (Beckman Coulter, USA) to quantify CD4^+^ and CD8^+^ T cells from 1 × 10^5^ acquired cells.

### 2.7 Enzyme-Linked Immune Absorbent Spot assay

Two weeks after the booster vaccination, immunized mice were euthanized, and their splenocytes were isolated. Splenocyte suspensions were prepared at a concentration of 1 × 10^5^ cells per well and cultured with 2 μg/mL of rCPA-CTD in 96-well plates for 24 hours. Splenocytes isolated from PBS-immunized control mice were stimulated with the same rCPA-CTD. IFN-γ and IL-4 secretion were detected using a mouse IFN-γ and IL-4 ELISpot kit (Dakewe Biotech, China) according to the manufacturer’s instructions. Cytokines were captured by specific monoclonal antibodies coated on the PVDF membrane of the ELISPOT plate. After cell lysis and removal, the captured cytokines were bound to biotin-labeled monoclonal antibodies, followed by incubation with horseradish peroxidase (HRP)-conjugated streptavidin. Upon substrate addition, specific spots formed on the PVDF membrane were quantified using an Enzyme Linked Spots (AID, Germany).

### 2.8 Challenge of immunized mice

Three weeks after the booster vaccination, immunized mice were challenged with *C. perfringens* alpha-toxin to induce enterotoxemia or with live bacteria to induce gas gangrene. For the toxin challenge, crude concentrated alpha-toxin was diluted to doses of 1, 5, 10, or 20 LD_100_ in a final volume of 0.5 mL per mouse. Each dose (0.5 mL per mouse) was administered intraperitoneally (IP) to groups of 5 mice. For the bacterial challenge, live *C. perfringens* type A strain HLJ-A5 was resuspended in PBS to a final volume of 0.2 mL containing 5 × 10^8^ or 5 × 10^9^ CFU. Each concentration (0.2 mL per mouse) was injected intramuscularly (IM) into the hind leg of 5 mice per group. Animal survival was monitored daily for 14 days following the challenge.

### 2.9 Histopathologic observation

For the toxin challenge, mice immunized with CPA-CTD mRNA vaccine and challenged with 20 LD_100_ of *C. perfringens* alpha-toxin, along with untreated healthy mice, were euthanized prior to dissection. Additionally, PBS-immunized control mice that succumbed to 1 LD_100_ alpha-toxin challenge were dissected post-mortem. Organs (intestine, lung, spleen, kidney, liver, and heart) were collected from all three groups. For the bacterial challenge, mRNA-vaccinated mice challenged with two doses of live *C. perfringens* (5 × 10^8^ or 5 × 10^9^ CFU) were euthanized one week post-challenge, and leg muscles from the infection site were isolated. All collected tissues were fixed in 4% paraformaldehyde for 12 hours, embedded in paraffin, cut into sections, and stained with hematoxylin and eosin (H&E) for histopathological analysis.

### 2.10 *In vivo* neutralization assay

The *in vivo* neutralization assay was conducted to measure alpha-toxin neutralizing antibody titer (NAT) in serum from immunized calves, immunized pregnant cows, and newborn calves, as well as in colostrum from immunized pregnant cows. Samples were serially diluted two-fold with PBS from a 1:2 dilution, with each dilution at 100 μL. Each dilution was mixed with PBS containing crude alpha-toxin at 1 LD_100_ per mouse, reaching a final volume of 300 μL, and incubated at 37°C for 1 hour. The mixtures were then intraperitoneally injected into groups of 4 unimmunized healthy mice each. NAT was defined as the highest serum dilution ensuring complete survival of a group of mice. To further assess the neutralizing capacity of immune serum, 100 μL of serum collected from immunized calves 3 weeks post-booster vaccination was mixed with crude concentrated alpha-toxin at 10 or 15 LD_100_ per mouse, diluted with PBS to a final volume of 300 μL, and incubated at 37°C for 1 hour. These mixtures were intraperitoneally injected into groups of 10 mice each, and survival rates were recorded over 14 days.

### 2.11 Statistical analysis

GraphPad Prism 8 software was used for statistical analysis. All data were expressed as the mean ± SD. Differences between groups were examined for statistical significance using a mixed-effects analysis or a one-way analysis of variance (ANOVA) with Tukey’s multiple comparison post-test. The asterisks in the figures indicate significant differences, with P < 0.05 (*P < 0.05; **P < 0.01; ***P < 0.001; ****P < 0.0001 ns, not significant).

## 3 Results

### 3.1 Preparation and characterization of a CPA-CTD mRNA-LNP vaccine

**Figure 2:**
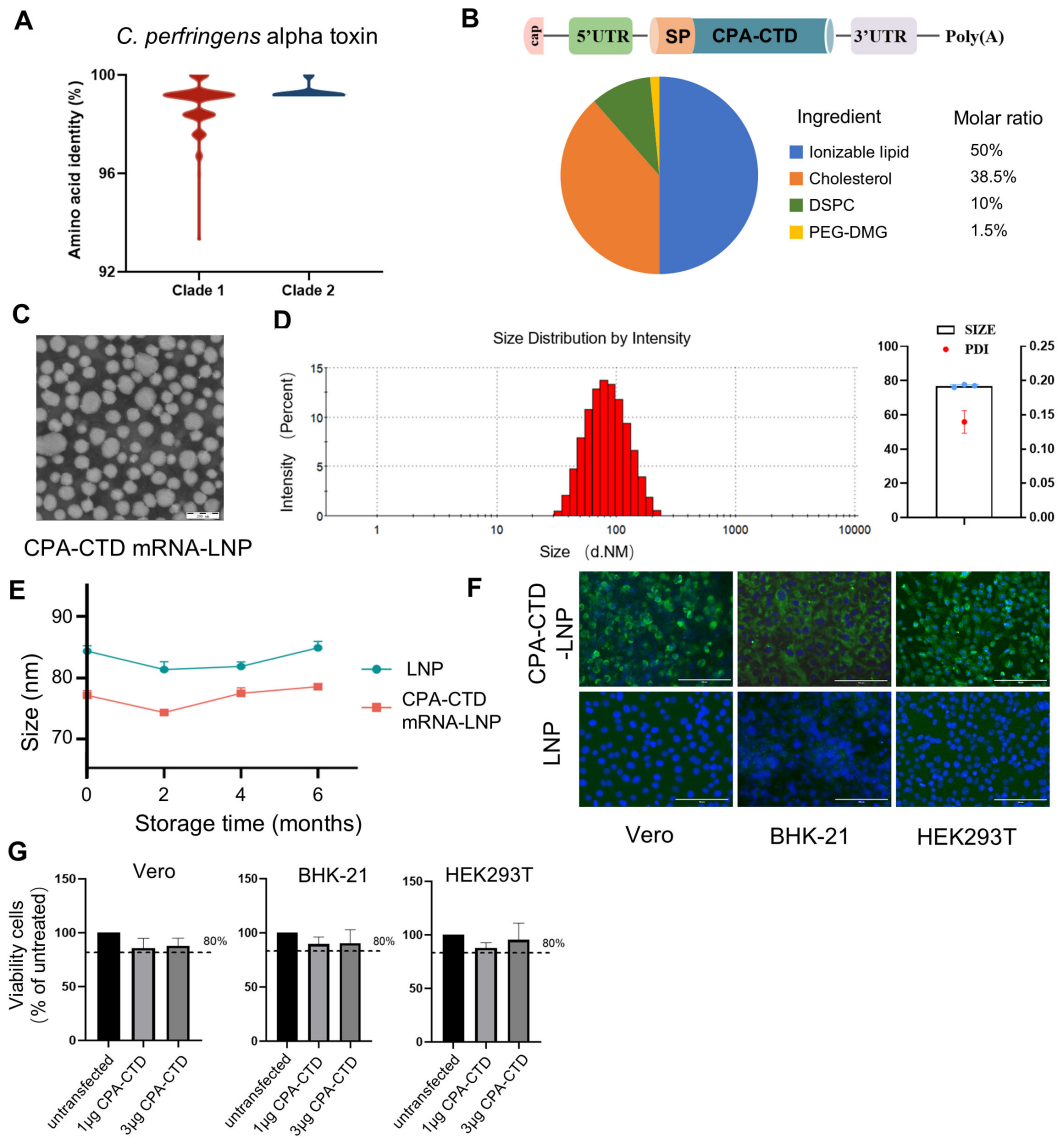
Production and characteristics of CPA-CTD mRNA vaccine candidate. **(A)** Amino acid sequence identity of the CPA-CTD from our mRNA construct compared to 138 *C. perfringens* strains across two clades, Clade 1 represents the CPA-CTD produced by *C. perfringens* type A, while Clade 2 represents the CPA-CTD produced by *C. perfringens* types B through E. **(B)** Schematic representation of CPA-CTD mRNA and mRNA-LNP formulation used throughout the study. **(C)** The images of LNP-encapsulated CPA-CTD mRNA by transmission electron microscopy. Scale bar, 200 nm. **(D)** Representative particle size and polydispersity index graph of mRNA-LNP. **(E)** Stability of mRNA-LNP particle size after storage at -20 °C for varying durations. **(F)** Detection of mRNA-LNP expression in Vero, BHK-21, and HEK293T cells by IFA using a mouse CPA-CTD-specific monoclonal antibody. **(G)** The cellular viability was assessed using a CCK-8 kit following the *in vitro* transfection of mRNA.

To develop an mRNA vaccine targeting *C. perfringens* alpha-toxin for preventing gas gangrene and enterotoxemia, we selected the CPA-CTD gene encoding the C-terminal domain (amino acids 247–370) of the alpha-toxin as the target, given that recombinant proteins of this fragment are non-toxic while retaining potential immunogenicity. To assess the sequence conservation of this vaccine target, we conducted a comparative analysis of CPA-CTD sequences from 138 representative *C. perfringens* strains in GenBank, revealing minimum, median, and mean amino acid similarities of 93.3%, 100%, and 98.9%, respectively, to our construct (**Figure 2A**, **Supplementary Figure S1**). To evaluate expression of the designed immunogen in eukaryotic systems, a codon-optimized plasmid encoding CPA-CTD was transfected into HEK293T cells, with efficient expression confirmed by IFA and Western blot (**Supplementary Figure S2**).

The CPA-CTD mRNA, fused with a tpA signal peptide and essential elements, was encapsulated into LNP and validated through quality control parameters (**Figure 2B**). Transmission electron microscopy confirmed uniform size distribution (**Figure 2C**). Physicochemical characterization of the CPA-CTD mRNA-LNP revealed an average size of 76.9 nm and a polydispersity index (PDI) of 0.139 (**Figure 2D**). The particle sizes of CPA-CTD mRNA-LNP formulations remained stable and consistent over six months of storage at 20°C (**Figure 2E**). Following transfection into BHK-21, HEK293T, and Vero cells, CPA-CTD mRNA-LNP showed efficient CPA-CTD expression and secretion (**Figure 2F**). Notably, *in vitro* expression of CPA-CTD mRNA-LNP did not compromise cell viability (**Figure 2G**), supporting its safety as an intramuscular immunogen in animals.

### 3.2 CPA-CTD mRNA vaccine elicits robust humoral and cellular immune responses in mice

**Figure 3:**
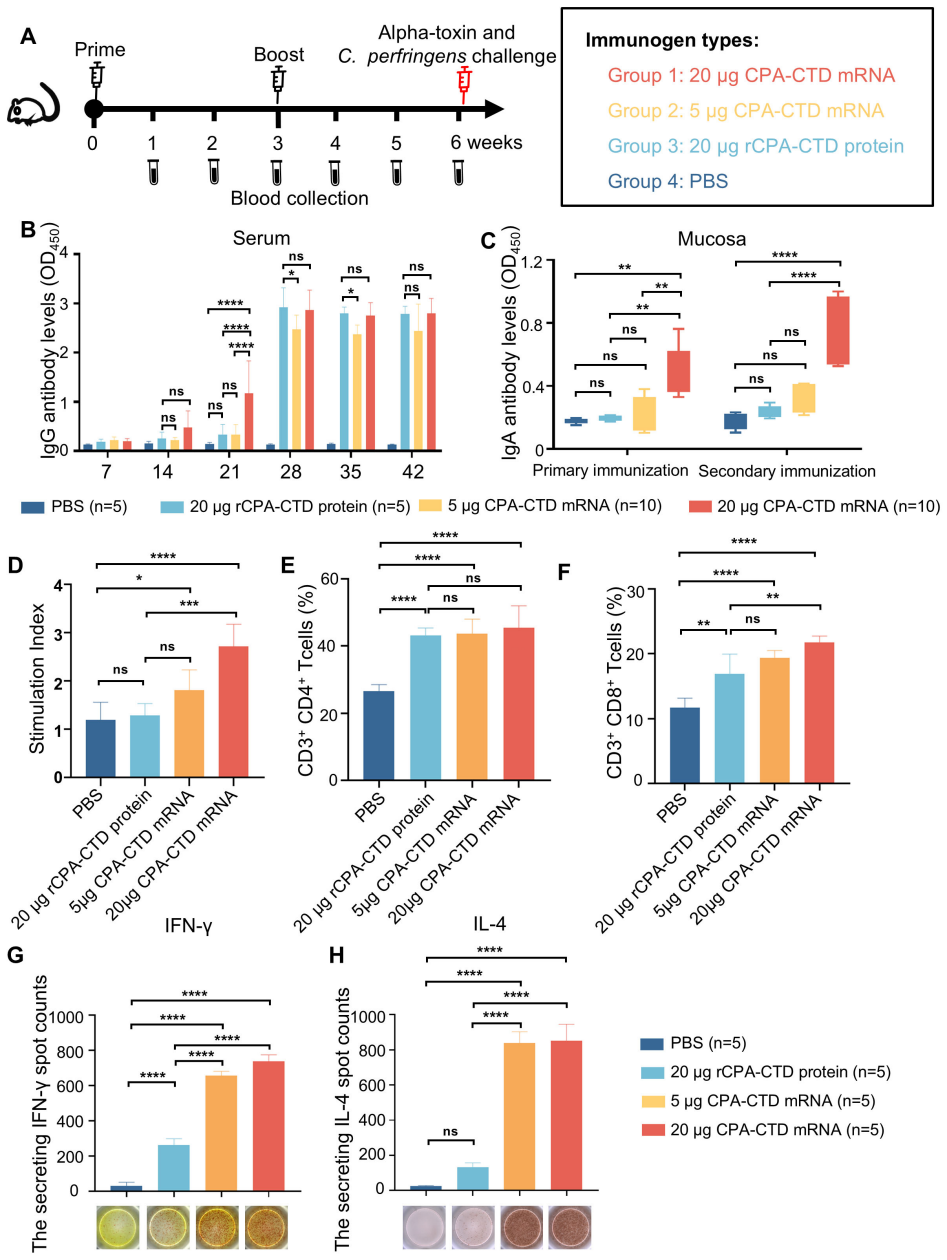
Robust immune responses in mice induced by the CPA-CTD mRNA vaccine. **(A)** Schematic diagram of immunization and challenge experiments in mice. BALB/c mice were immunized with two doses of CPA-CTD mRNA vaccine, rCPA-CTD protein, or PBS control, and boosted with an equal dose 3 weeks later. Serum samples were collected at the indicated time points post-immunization. **(B)** Anti-CPA-CTD IgG levels in sera from immunized mice determined by ELISA. **(C)** Anti-CPA-CTD IgA levels in feces from immunized mice determined by ELISA. **(D)** rCPA-CTD-specific lymphocyte proliferation assay. The lymphocytes were stimulated with rCPA-CTD for 48 h, and the proliferation rate was measured using a CCK-8 kit. The relative stimulation index was calculated as the ratio of the average OD value of antigen-stimulated wells to that of unstimulated wells. **(E, F)** The frequencies of CD3^+^ CD4^+^and CD3^+^ CD8^+^ T cells in mice after immunization. The splenic lymphocytes of mice were isolated and characterized by flow cytometry to enumerate CD4^+^and CD8^+^T cells. **(G, H)** The spot counts of IFN-γ and IL-4 secreting splenocytes in ELISpot assays. Splenocyte were stimulated by recombinant rCPA-CTD protein. The results are presented as the mean ± standard error of the mean (SEM). Differences between groups using one-way ANOVA are shown. *p < 0.05, **p < 0.01, ***p < 0.001, ****p < 0.0001. ns, not significant.

To assess the immunogenicity of CPA-CTD mRNA vaccine, BALB/c mice underwent two intramuscular immunizations three weeks apart (**Figure 3A**). Recombinant CPA-CTD, expressed and purified from *E. coli* (**Supplementary Figure S3**), was used as a subunit vaccine control and administered concurrently with the mRNA vaccine (**Figure 3A**). Following immunization, mice did not exhibit abnormal body weight fluctuations or overt adverse reactions, indicating a favorable safety profile for the vaccine (**Supplementary Figure S4A**). After the booster immunization, both the CPA-CTD mRNA vaccine and rCPA-CTD groups exhibited a significant increase in specific IgG antibodies, with the mRNA vaccine eliciting a pronounced dose-dependent IgG response. Notably, statistical analysis revealed the high-dose group (20 μg) exhibited more than threefold higher IgG levels than the subunit vaccine group 14–21 days after the initial immunization, indicating a faster induction (**Figure 3B**). Fecal analysis revealed that alpha-toxin-specific IgA levels in the CPA-CTD mRNA vaccine group were significantly higher than those in the rCPA-CTD and PBS control groups, suggesting a robust mucosal immune response (**Figure 3C**) At 42 days post-vaccination, splenic lymphocytes from the CPA-CTD mRNA-LNP group, stimulated *in vitro* with rCPA-CTD (10 μg/mL), showed a proliferation index substantially higher than that in the rCPA-CTD and PBS control groups, with the 20 μg dose group outperforming the 5 μg dose group (**Figure 3D**). The percentages of CD3^+^ CD4^+^ and CD3^+^ CD8^+^ T cells in both the CPA-CTD mRNA vaccine and rCPA-CTD groups were significantly elevated compared to the PBS control group (**Figures 3E, F**). Furthermore, IFN-γ and IL-4 levels produced by lymphocytes in the CPA-CTD mRNA vaccine group were significantly higher than those in the rCPA-CTD and PBS control groups, with the 20 μg dose group surpassing the 5 μg dose group (**Figures 3G, H**). Collectively, these data, supported by comprehensive statistical comparisons across all humoral and cellular readouts (**Figures 3B–H**), indicate that the CPA-CTD mRNA vaccine, particularly at the higher 20 μg dose, induces stronger and more comprehensive immune responses than the rCPA-CTD subunit vaccine.

### 3.3 CPA-CTD mRNA vaccine protects mice against enterotoxemia induced by lethal doses of *C. perfringens* alpha-toxin

**Figure 4:**
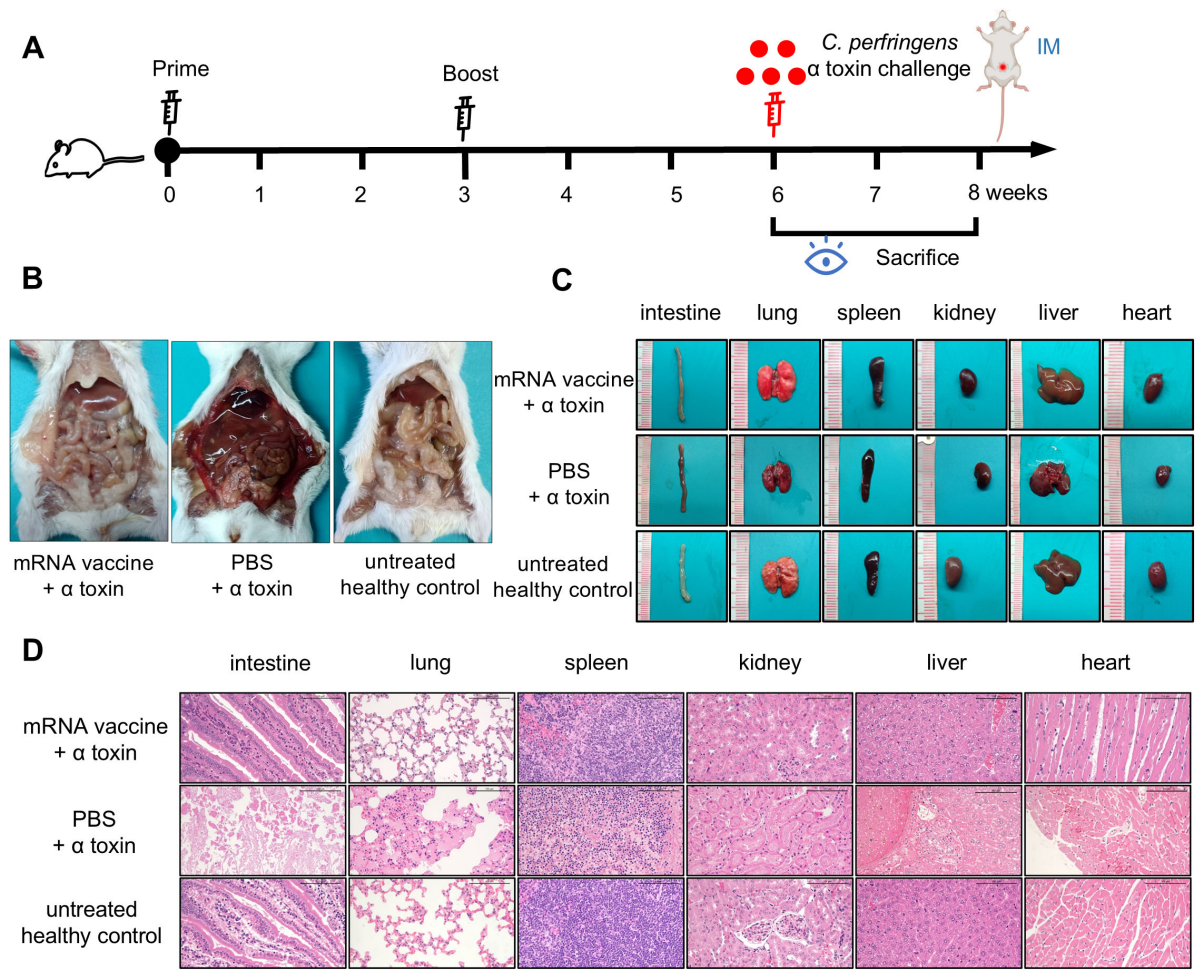
Immune protection of mice immunized with CPA-CTD mRNA vaccine against alpha-toxin challenge. **(A)** Schematic diagram of alpha-toxin challenge in immunized mice. Three weeks post-booster immunization, the mice were intraperitoneally challenged with alpha-toxin. **(B)** Gross lesions in peritoneal cavity of challenged mice. **(C)** A thorough examination of pathological alterations after the challenge across various organs, including the intestinal, lung, spleen, kidney, liver, and heart. **(D)** Histopathological alterations in murine organs were examined using Hematoxylin and Eosin (H&E) staining. Scale bar, 100 μm.

To evaluate the protective efficacy of the CPA-CTD mRNA vaccine against enterotoxemia induced by *C. perfringens* alpha-toxin, immunized mice underwent a challenge with varying doses of toxin via intraperitoneal injection three weeks after booster vaccination (**Figure 4A**). The toxin used for the challenge consisted of crude concentrated alpha-toxin, with LD_100_ determined in mice (**Supplementary Figure S5**). Results indicated that, under a 1 LD_100_ toxin challenge, all PBS control mice died, demonstrating absence of protection. At 1 LD_100_, 5 LD_100_, and 10 LD_100_ toxin challenges, both 5 μg and 20 μg CPA-CTD mRNA vaccines, along with the rCPA-CTD subunit vaccine, achieved 100% protection in mice. However, at a 20 LD_100_ toxin challenge, the 5 μg CPA-CTD mRNA group exhibited a protection rate of 40%, the rCPA-CTD subunit vaccine group reached 80%, and the 20 μg CPA-CTD mRNA group maintained 100% (**Table 1**). In terms of clinical manifestations, vaccine-immunized mice exhibited a transient body weight reduction of approximately 5% following alpha toxin challenge, with complete recovery within two weeks, and no other abnormal symptoms were observed (**Supplementary Figure S4B**). These findings demonstrate that the CPA-CTD mRNA vaccine effectively protects mice against lethal toxin challenges, with efficacy showing dose dependence.

**Table 1:** Immune protection of mice vaccinated with CPA-CTD mRNA vaccine and rCPA-CTD against lethal challenge of *C. perfringens* alpha-toxin.

Vaccine	Dose (μg)	Survival rate of mice after challenge (survival/total)			
		1 LD_100_	5 LD_100_	10 LD_100_	20 LD_100_
PBS	0	0(0/5)	ND	ND	ND
CPA-CTD mRNA	5	100% (5/5)	100%(5/5)	100%(5/5)	40%(2/5)
CPA-CTD mRNA	20	100%(5/5)	100%(5/5)	100%(5/5)	100%(5/5)
rCPA-CTD	20	100%(5/5)	100%(5/5)	100%(5/5)	80%(4/5)

To further confirm protective effects, gross lesions and histopathological changes in challenged mice were examined using necropsy and H&E staining. PBS control mice displayed typical enterotoxemia symptoms, including significant abdominal hemorrhage, severe colonic tissue fragmentation, and hemorrhagic spots and necrotic foci in organs such as liver, kidneys, and lungs. In contrast, CPA-CTD mRNA-immunized mice exhibited no abnormal clinical signs post-challenge. Necropsy revealed abdominal cavities similar to those of healthy mice, with no hemorrhage observed, and no significant gross lesions detected in any organs (**Figures 4B, C**). Histopathological analysis supported these findings: PBS control mice showed complete destruction of intestinal mucosa and submucosa, with only partial crypt and villi outlines remaining; some lung alveolar cavities contained serous exudate; spleen structure appeared blurred, with red pulp showing erythrocyte hemolysis and white pulp exhibiting lymphocyte dissociation and reduction; kidney tubular epithelial cell nuclei appeared condensed or lysed; liver displayed congestion in central and portal veins; and heart showed mild epicardial and interstitial edema, right ventricular dilation, and ventricular wall thinning (**Figure 4D**). In contrast, CPA-CTD mRNA-immunized mice exhibited no significant histopathological damage in these tissues (**Figure 4D**). These results demonstrate that the CPA-CTD mRNA-LNP vaccine robustly protects mice against alpha-toxin-induced enterotoxemia.

### 3.4 The protective efficacy of CPA-CTD mRNA vaccines against gas gangrene caused by *C. perfringens* infection

**Figure 5:**
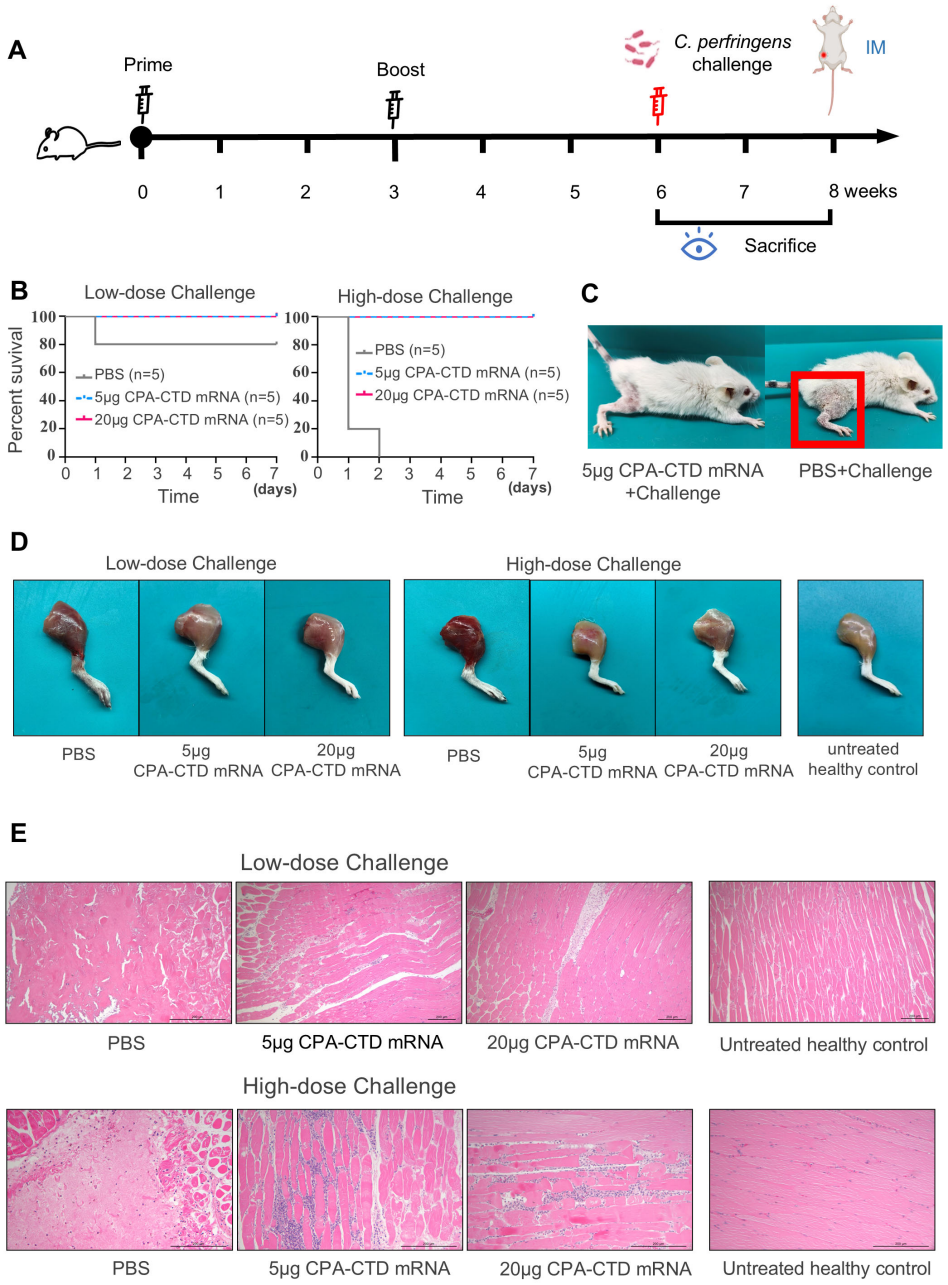
Immune protection of mice immunized with CPA-CTD mRNA vaccine against *C. perfringens* infection. Schematic diagram of *C. perfringens* challenge in immunized mice. Three weeks post-booster immunization, the mice were intramuscularly challenged with *C. perfringens.***(B)** Survival rates of immunized mice challenged with two distinct doses of *C. perfringens*. **(C)** Clinical symptoms in PBS control mice challenged with *C. perfringens*: muscle congestion, swelling, and lameness. **(D)** Gross lesions in the leg muscles of mice challenged with two distinct doses of *C. perfringens***(E)** Histopathological alterations in the leg muscles of mice challenged with two distinct doses of **(*C*)***perfringens* using H&E staining. Scale bar, 200 μm.

To evaluate the protective efficacy of the CPA-CTD mRNA vaccine against gas gangrene induced by *C. perfringens* infection, immunized mice were challenged with *C. perfringens* at high (5 × 10^9^ CFU/mouse) and low (5 × 10^8^ CFU/mouse) doses injected into leg muscles (**Figure 5A**). Results showed that high-dose infection led to 100% mortality in PBS-immunized mice, while low-dose infection resulted in an 20% mortality rate (**Figure 5B**), with surviving mice exhibiting muscle congestion, swelling, and lameness (**Figure 5C**). In contrast, mice immunized with 5 μg and 20 μg CPA-CTD mRNA survived both doses (**Figure 5B**), showing only mild lameness. Clinically, immunized mice exhibited an average body weight reduction of approximately 6% following challenge (**Supplementary Figure S4C**), accompanied by mild lameness, and returned to normal within two weeks.

To compare macroscopic and histopathological changes between CPA-CTD mRNA-immunized mice and PBS control mice, three mice per group were randomly selected, euthanized, and dissected at 3 days post-challenge. Leg muscles of PBS-immunized mice displayed significant congestion, hemorrhage, swelling, and necrosis. In contrast, leg muscles of CPA-CTD mRNA-immunized mice exhibited mild macroscopic lesions (slight congestion and swelling), markedly less severe than in the PBS group (**Figures 5C, D**). Histopathological examination further revealed severe lesions in leg muscles of PBS-immunized mice, including muscle fiber fragmentation and necrosis, fibrinous exudation, and hemorrhage. Conversely, leg muscles of CPA-CTD mRNA-immunized mice showed only minimal inflammatory cell infiltration, with lesion severity significantly reduced compared to the PBS group (**Figure 5E**). These findings indicate that the CPA-CTD mRNA vaccine provides robust protection against *C. perfringens*-induced gas gangrene in mice, preventing mortality and markedly reducing symptoms and pathology.

### 3.5 Evaluation of the immunogenicity by CPA-CTD mRNA vaccine in cattle

**Figure 6:**
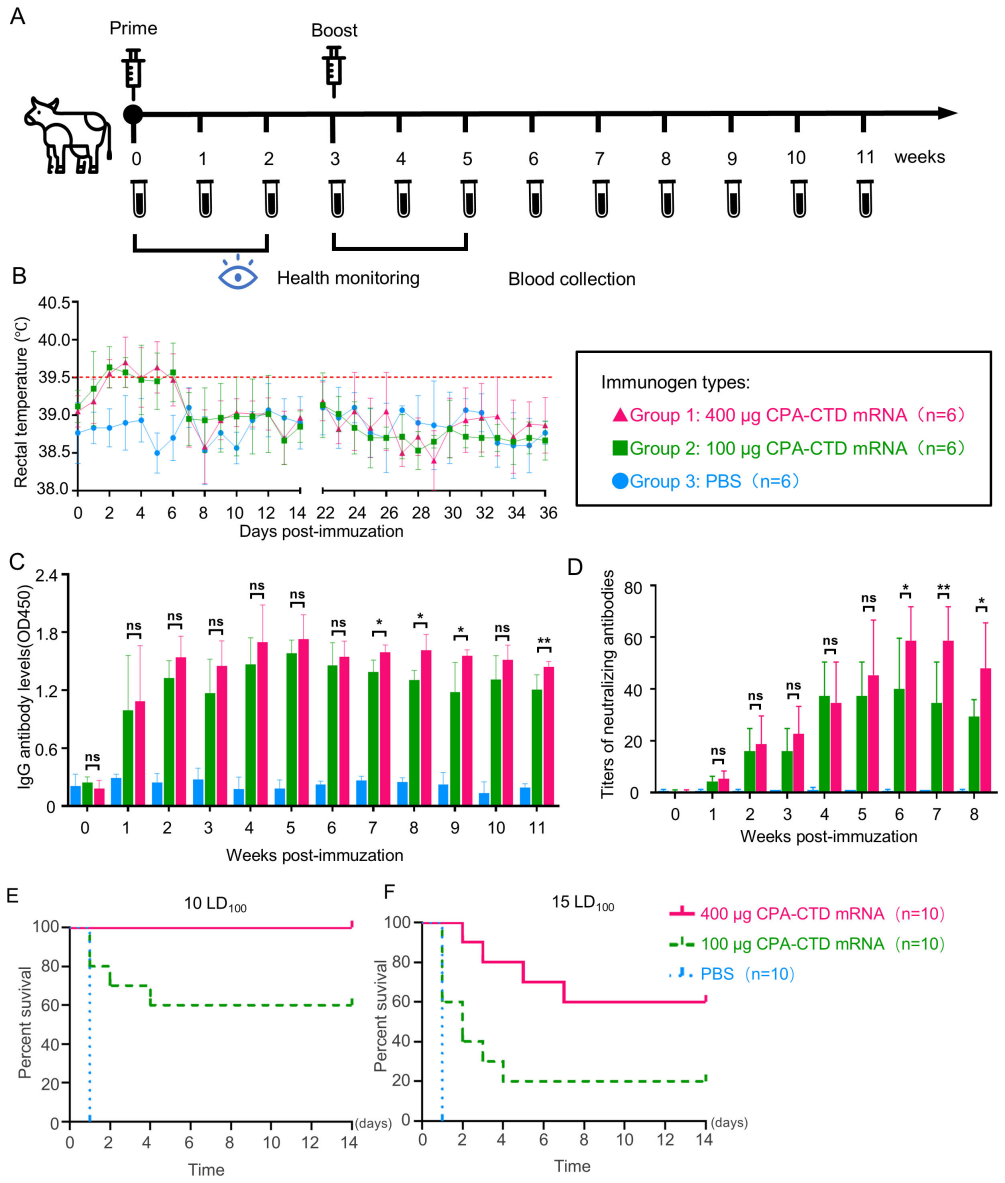
Protective immune responses induced by the CPA-CTD mRNA vaccine in calves. **(A)** Schematic diagram of calf grouping, immunization, and blood collection. **(B)** Temperature measurements of the calves after immunizations. **(C)** CPA-CTD-specific IgG in sera of immunized calves induced by CPA-CTD mRNA vaccine. **(D)** The NAT in sera of immunized calves induced by CPA-CTD mRNA vaccine. **(E, F)** Survival rates of mice injected with mixtures of bovine sera collected three weeks after booster immunizations and 10 LD_100_**(E)** or 15 LD_100_**(F)** alpha-toxin. *p < 0.05, **p < 0.01. ns, not significant.

To assess the ability of the CPA-CTD mRNA vaccine to induce protective immune responses in cattle, 6-month-old calves (6 per group) received two vaccinations at a 3-week interval (**Figure 6A**). Safety evaluation revealed no abnormalities in injection sites, appetite, or overall health at 100 μg or 400 μg doses, with only transient body temperature increases (not exceeding 1°C above baseline) observed in a few calves, which resolved rapidly (**Figure 6B**). These findings suggest high safety of the vaccine in calves.

Vaccine efficacy was evaluated by measuring alpha-toxin-specific IgG and neutralizing antibody levels in calf serum. After the primary immunization, IgG levels increased significantly in both dose groups, peaked 2–3 weeks after the booster, and then remained stable for 8 weeks (**Figure 6C**), indicating durable humoral responses. *In vivo* neutralization assays showed that primary immunization induced protective antibodies, with the 100 μg dose group achieving an average NAT of 1:16, while the 400 μg dose group exhibited an NAT of 1:22.7. After booster immunization, the NAT against the toxin in immune sera was significantly increased in both groups. Statistical analysis confirmed that the peak NAT in the high-dose group was modestly but significantly higher and approximately 1.37-fold greater than that in the low-dose group, although both doses induced high neutralizing titers and robust neutralizing activity against alpha-toxin (**Figure 6D**). To further confirm the neutralizing efficacy of immune sera against alpha-toxin, mice were injected with mixtures of 10 LD_100_ or 15 LD_100_ alpha-toxin and sera collected at three weeks after booster immunization. When the toxin dose was 10 LD_100_, serum from the 400 μg immunized group protected all mice from mortality, while serum from the 100 μg immunized group protected 60% of mice (**Figure 6E**). When the toxin dose was increased to 15 LD_100_, the protection rate in the 100 μg serum group dropped to 20%, while the 400 μg serum group still protected 60% of mice (**Figure 6F**). Taken together, these results, along with the statistical comparisons between dose groups (**Figures 6C–F**), confirm that the CPA-CTD mRNA vaccine elicits potent, dose-dependent neutralizing antibody responses in calves and has strong potential for preventing alpha-toxin-related diseases caused by *C. perfringens*.

### 3.6 Passive immunity of newborn calves conferred by immunization of pregnant cows with CPA-CTD mRNA vaccine

**Figure 7:**
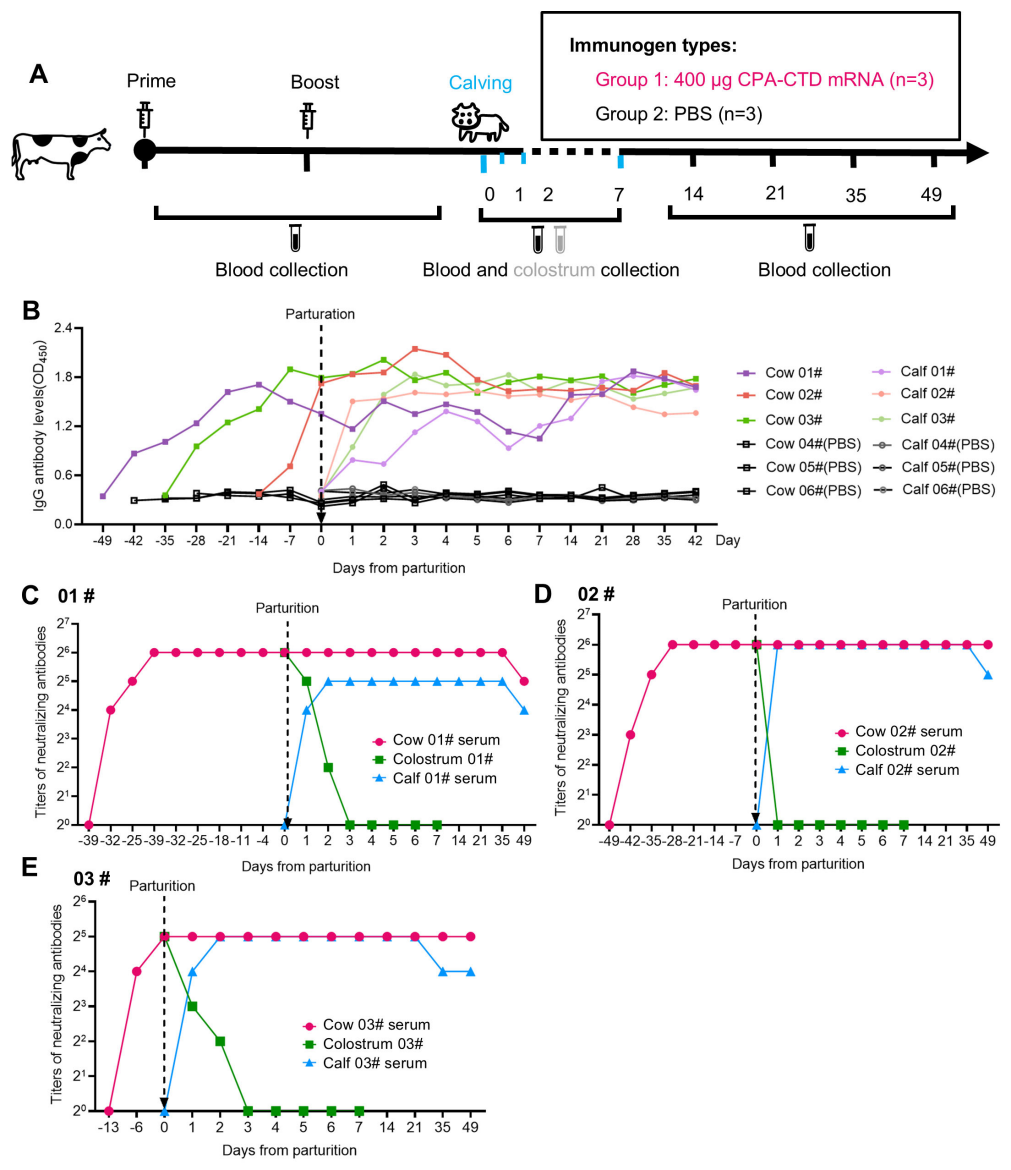
Passive immunity for newborn calves through immunization of pregnant cows with CPA-CTD mRNA. **(A)** Schematic diagram of immunization, parturition, and sample collection from pregnant cows and calves. **(B)** Dynamics of specific IgG antibodies in the serum of pregnant cows and newborn calves. **(C-E)** Measurement of alpha-toxin NAT of sera and colostrums from cows and sera from newborn calves.

To evaluate passive immunity conferred to newborn calves via CPA-CTD mRNA vaccination of pregnant cows, three cows (designated 01#, 02#, and 03#) were vaccinated prior to calving: 01# and 02# received two doses, while 03# received one dose due to misestimated gestation timing. Three additional cows received two PBS doses as controls (**Figure 7A**). Post-vaccination, these cows developed high levels of alpha-toxin-specific IgG antibodies (**Figure 7B**), whereas no specific antibodies were detected in serum or colostrum of PBS control cows (data not shown). In newborn calves, serum IgG levels post-colostrum intake matched those of their dams (**Figure 7B**). *In vivo* neutralization assays revealed that, the average NAT in serum of the three vaccinated cows on the day of calving reached 1:53.3, with colostrum NAT ranging from 1:32 to 1:64 (**Figures 7C–E**). After colostrum consumption, calf serum NAT averaged 1:42.7, remaining between 1:32 and 1:64 for 35 days post-birth (**Figures 7C–E**), while no neutralizing antibodies were detected in control calves’ serum (data not shown). These findings demonstrate that vaccinating pregnant cows with CPA-CTD mRNA effectively transfers protective antibodies via colostrum, conferring passive immunity against *C. perfringens* alpha-toxin to newborn calves.

## 4 Discussion

Compared to traditional recombinant protein vaccines, mRNA-LNP vaccine technology offers advantages in rapid production, ease of modification, and the ability to express multiple antigens, while inducing sustained immunity without additional adjuvants. Although subunit vaccines have been explored for *C. perfringens* for years, practical applications remain scarce, highlighting the need for novel platforms such as mRNA. This study pioneers the application of mRNA-LNP technology to develop a candidate vaccine targeting *C. perfringens* toxin, aimed at preventing lethal diseases caused by this pathogen (7, 18). This endeavor not only opens new avenues for *C. perfringens* vaccine research but also provides a significant reference for the use of mRNA technology in preventing bacterial diseases.

The universality of a vaccine is a critical factor in its development and application. The CPA-CTD mRNA vaccine designed in this study is based on the highly conserved C-terminal domain of the *C. perfringens* alpha-toxin. This sequence exhibits high homology with 138 published alpha-toxin amino acid sequences covering toxin types A-E. As alpha-toxin is an exotoxin secreted by all *C. perfringens* toxin types, its sequence conservation makes it an ideal vaccine target. This conservation not only targets type A strains, which predominantly secrete alpha-toxin, but may also provide auxiliary protection against complex tissue damage caused by the synergistic action of multiple toxins in other toxin types (30), thereby expanding the potential scope of the vaccine’s application. Furthermore, the expression capability of this mRNA vaccine in various cell lines further supports its universality. Experimental results demonstrate that CPA-CTD mRNA can be effectively expressed in human (HEK293T), monkey (Vero), and murine (BHK-21) cells, indicating its potential for cross-species expression. However, attempts to express the vaccine in bovine MDBK cells did not yield significant antigen expression, likely due to the inherently low transfection efficiency of MDBK cells rather than a flaw in the vaccine design. These differences in expression across cell lines suggest the need for further optimization of transfection conditions or evaluation of alternative bovine cell models in future studies to fully validate the vaccine’s applicability. Nonetheless, the current expression data already provide a solid foundation for the broad application potential of the CPA-CTD mRNA vaccine and its subsequent validation.

The speed and type of immune response induced by vaccines are critical for effectively preventing *C. perfringens* infection. This study found that the CPA-CTD mRNA vaccine rapidly and efficiently induces specific IgG antibodies in mice by the second week post-immunization, outperforming the rCPA-CTD subunit vaccine with a dose-dependent effect. This swift antibody response is vital for emergency prevention during *C. perfringens* outbreaks or high-risk scenarios. Since alpha-toxin primarily causes disease via the intestinal mucosa, mucosal immunity may play a key role in prevention (31, 32). Data indicate that the CPA-CTD mRNA vaccine elicits higher levels of alpha-toxin-specific IgA antibodies than the subunit vaccine, highlighting its advantage in activating mucosal immunity. In addition, subsequent to the booster immunization, the high-dose mRNA vaccine formulation induced a more robust antibody response, with titers that were significantly elevated relative to the subunit vaccine group. Furthermore, the vaccine activates CD4^+^ and CD8^+^ T cells in immunized mice, with IL-4 and IFN-γ production detected via ELISPOT, indicating Th1 and Th2 immune responses. Collectively, compared to the subunit vaccine, the CPA-CTD mRNA vaccine more comprehensively and rapidly triggers multiple immune responses in mice.

Alpha-toxin is a pivotal virulence factor in *C. perfringens* infection pathology, predominantly driving enterotoxemia and gas gangrene. To evaluate the protective efficacy of the CPA-CTD mRNA vaccine against alpha-toxin, we developed mouse models of enterotoxemia and gas gangrene that simulate natural infection. This study is the first to utilize both models simultaneously for immunological assessment. In the enterotoxemia model, while the CPA-CTD subunit vaccine is highly immunogenic, it did not provide complete protection against a 20 LD_100_ dose. In contrast, mice in the 20 μg mRNA vaccine group were 100% protected, with no evident pathological changes observed in challenged mice. These results demonstrate the strong protective efficacy of the mRNA vaccine against enterotoxemia.

For the gas gangrene model, we employed two challenge doses: a high dose (fully lethal) and a low dose (inducing typical symptoms and pathology without complete lethality) to comprehensively assess the mRNA vaccine’s performance. The mRNA vaccine protected mice from both doses, preventing survival loss and the characteristic symptoms and pathology of gas gangrene. Although mild swelling and lameness were observed in some immunized mice post-challenge, likely due to inflammation from extensive bacterial stimulation of muscle tissue, histopathological analysis revealed only minor inflammatory cell infiltration, which did not compromise overall protection. Collectively, the CPA-CTD mRNA vaccine demonstrated robust protective efficacy in both disease models, underscoring its exceptional potential for *C. perfringens* infection control.

Type A *C. perfringens*, by secreting alpha-toxin, is a primary cause of bovine enterotoxemia, posing a severe threat (13, 33); thus, we evaluated the CPA-CTD mRNA vaccine’s immune protective efficacy in target animal cattle. We and others have confirmed that the C-terminal domain (CTD) of alpha-toxin confers protection against bovine enterotoxemia (7, 13, 34, 35). However, even with an exceptionally high dose (600 μg) of rCPA-CTD protein, no significant neutralizing antibodies were induced after initial immunization, reaching only a 1:40 titer post-booster (3). In contrast, the CPA-CTD mRNA vaccine developed in this study elicited neutralizing antibody titers of 1:16 and 1:22.7 in cattle at 100 μg and 400 μg doses, respectively, after the first immunization—consistent with the rapid immune response observed in mice—rising to 1:42.7 and 1:58.7 post-booster. This suggests that CPA-CTD mRNA triggers a faster and stronger immune response in cattle, underscoring its potential as a candidate vaccine for rapid, effective protection following a single dose. Although high neutralizing antibody titers were measured in the mouse model, we acknowledge a key limitation of this study: a direct, quantitative correlation between these titers and the degree of protection in cattle has not yet been established. Additionally, the protective antibody threshold required for full immunity in cattle remains undefined. Therefore, future studies should focus on direct challenge experiments in cattle to assess vaccine efficacy. It is also essential to establish a clear correlation between *in vitro* neutralization data (generated in both species) and *in vivo* protection levels. Defining this correlation is crucial for the rational development and evaluation of future vaccines. Furthermore, while neutralizing antibody levels increased with dose, the magnitude of improvement was modest, suggesting that a lower dose may suffice for adequate protection. Serum IgG antibodies in cattle remained elevated for two months post-booster, highlighting the importance of this sustained immune response for livestock vaccination. Future studies with larger sample sizes and broader vaccination scales could further evaluate dose effects and long-term efficacy.

Enterotoxemia caused by *C. perfringens* markedly reduces neonatal calf survival rates, frequently causing substantial economic losses in the cattle industry. To address this, we investigated the potential of the CPA-CTD mRNA vaccine to confer passive protection to newborn calves by immunizing pregnant cows. During this process, an unexpected finding emerged: due to a misestimated calving date, cow#03 delivered after a single immunization, yet its serum neutralizing antibody titer reached 1:32. This reinforces that a single dose can elicit a robust immune response, offering ranchers flexibility to adapt vaccination schedules to practical needs. Findings indicated that neutralizing antibodies induced in vaccinated pregnant cows were efficiently transferred to calves via colostrum, absorbed through the intestine, reaching a peak serum titer within two days and persisting for one month, which is critical for early calf protection. However, given the small sample size in this study, future research should expand the trial scale to confirm the consistency of immunization efficacy.

Vaccine safety is a prerequisite for its practical application, particularly for novel platforms such as mRNA-based vaccines. In the characterization phase of the CPA-CTD mRNA vaccine, we observed robust antigen expression in multiple cell types (HEK293T, BHK-21, and MDBK) with minimal impact on cell viability, indicating a favorable safety profile at the cellular level. In the mouse safety evaluation, no significant weight variations or adverse reactions were observed post-vaccination (**Supplementary Figure S4A**). Similarly, in cattle, no adverse effects on mental state, appetite, or injection site reactions were noted, though a mild transient increase in body temperature was observed during the first week, which resolved by the second week. However, the current safety assessment possesses inherent limitations. To comprehensively evaluate the safety profile of the CPA-CTD mRNA vaccine, future studies should incorporate dose-escalation trials in cattle, with a focus on key safety endpoints, including: (i) systemic inflammatory markers such as neutrophil count and platelet levels, (ii) hepatic function panels (e.g., ALT, AST), and (iii) histopathological analysis of the injection site and immune organs (e.g., lymph nodes, spleen). These evaluations, along with further dose optimization, are essential to inform the design of pivotal clinical trials.

Compared to traditional *C. perfringens* toxoid vaccines or recombinant subunit platforms, the LNP-encapsulated mRNA vaccine technology employed in this study demonstrates potential for eliciting robust neutralizing antibody responses that are comparable to, or even superior to, those of subunit vaccines (14, 16–18, 22). However, this emerging platform has specific limitations. The stability of mRNA vaccines, including the retention of formulation integrity and activity during long-term storage, remains a major challenge, often requiring more stringent cold-chain conditions than conventional vaccines (29). The production of LNP-mRNA formulations is complex and costly, which may hinder their widespread use in large-scale livestock immunization programs (24). Although our study shows promising immunogenicity in mice, the efficacy, safety, and durability of mRNA vaccines in target species such as cattle still require definitive validation through direct challenge experiments. Future research should focus on optimizing LNP formulations for improved stability, developing cost-effective production methods, and conducting comprehensive evaluations in target animals to facilitate the practical application of this technology.

In summary, this study presents the first design of an mRNA vaccine candidate targeting the alpha toxin of *C. perfringens*, which was comprehensively evaluated in experimental mouse and target cattle models. The vaccine demonstrated exceptional immune efficacy in preventing alpha-toxin-induced enterotoxemia and gas gangrene, highlighting its significant potential for controlling *C. perfringens*-related diseases.

## Data Availability

The datasets presented in this study can be found in online repositories. The names of the repository/repositories and accession number(s) can be found in the article/**Supplementary Material**.
