# Supplementary material for: A lipid nanoparticle encapsulated CPA-CTD mRNA vaccine provides protection against Clostridium perfringens-driven diseases
Source: Front Immunol. 2026 Jan 8;16:1748171. doi: 10.3389/fimmu.2025.1748171 (PMC12823533; doi:10.3389/fimmu.2025.1748171)
Supplement: Supplementary file 1 [file SupplementaryFile1.docx]

### Supplementary materials

**Fig. S1**


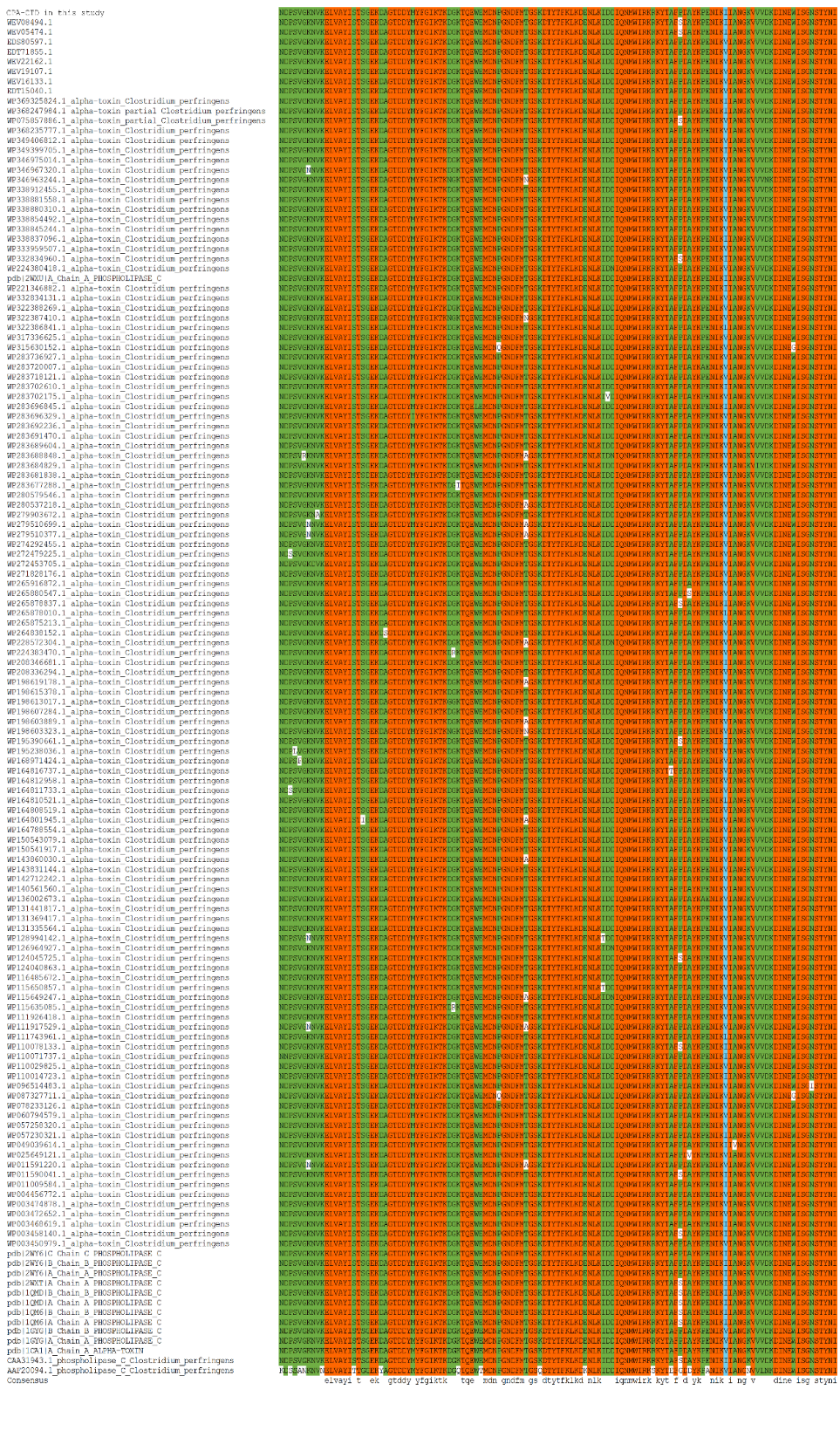


**Fig. S1 Multiple sequence alignment of *C. perfringens* CPA-CTDs available in GenBank.** The CPA-CTD sequence from *C. perfringens* HLJ-A5 strain (GenBank: PQ858782) was aligned with the CPA-CTD sequences of 138 *C. perfringens* strains from GenBank. Sequence identifiers are listed on the left, with fully conserved residues annotated below the alignment. Divergent residues are highlighted, revealing conserved and variable regions across the sequences.

**Fig. S2**


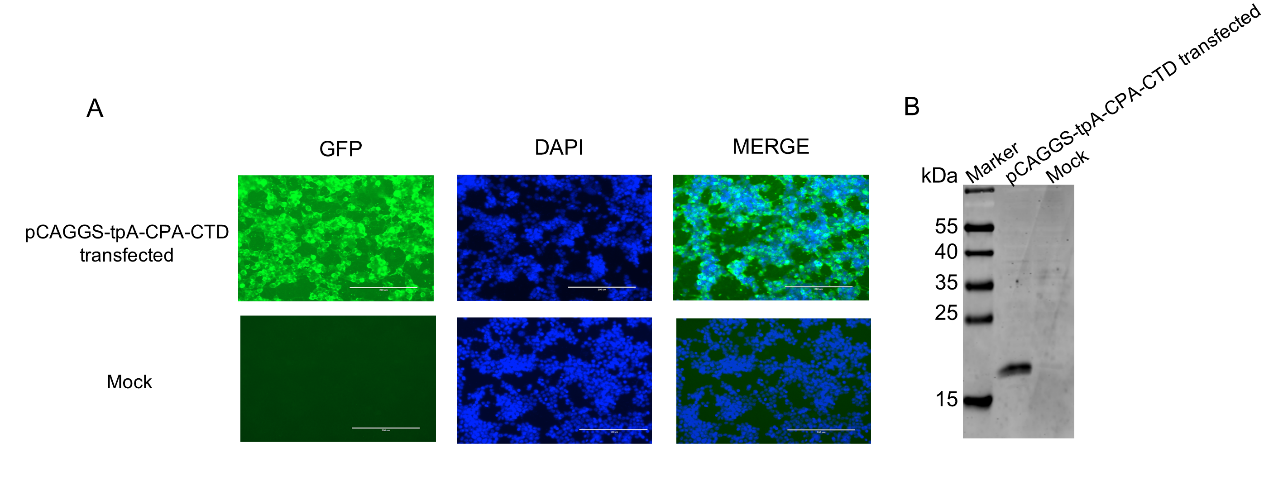


**Fig. S2 CPA-CTD protein expression in HEK293T cells.** The codon-optimized CPA-CTD gene fused with the tPA signal peptide sequence was cloned into the pCAGGS vector. HEK293T cells were transfected with the recombinant plasmid pCAGGS-tpA-CPA-CTD. At 48 h post-transfection, CPA-CTD protein expression was detected by IFA (A) and Western blotting (B) using a mouse CPA-CTD-specific monoclonal antibody.

**Fig. S3**


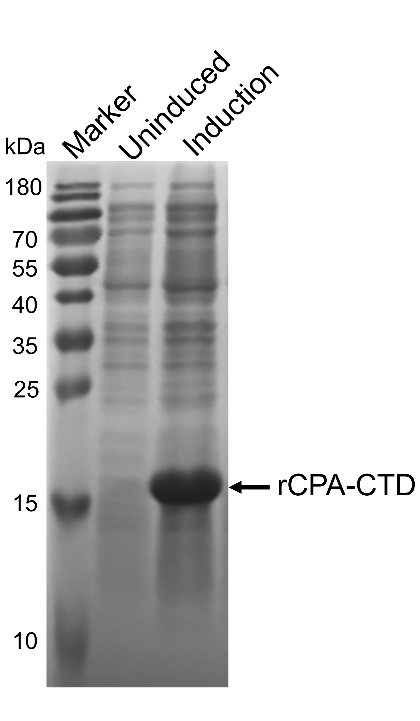


**Fig. S3 SDS-PAGE analysis of the rCPA-CTD expressed in *E. coli*.** The codon-optimized CPA-CTD sequence of HLJ-A5 strain (GenBank: PQ858782) was cloned into the pET-28a vector. The recombinant plasmid was transformed into Rosetta (DE3) competent E. coli cells (Merck, Darmstadt, Germany), and the expression of rCPA-CTD was induced with 0.5 mM IPTG at 30℃ for 6 h. An Ni2+ NTA affinity column (Qiagen, Hilden, Germany) was used to purify the soluble rCPA-CTD.

**Fig. S4**


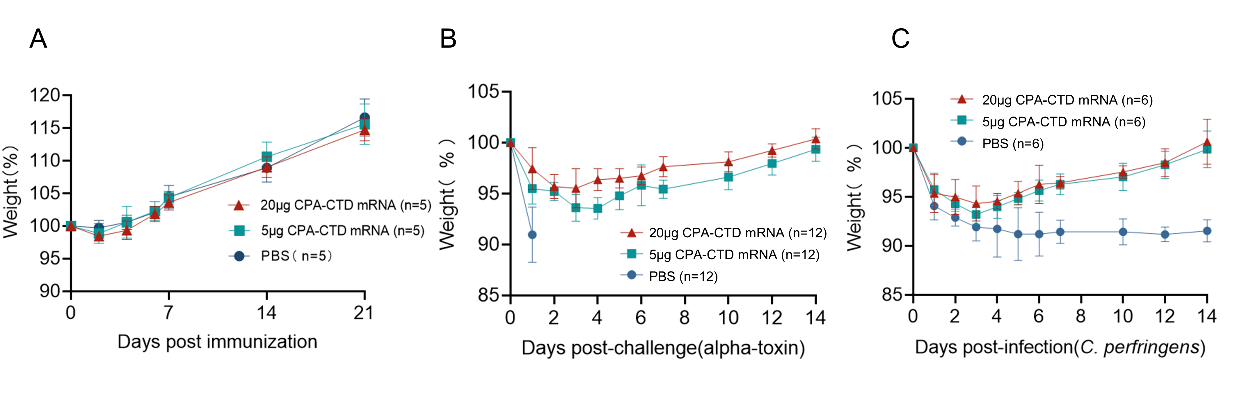


**Fig. S4 Body weight changes in mice following immunizations or challenges.** Five mice per group were randomly selected from those immunized with 20 µg/mouse or 5 µg/mouse CPA-CDT mRNA vaccine or PBS control to measure body weight changes after the initial immunization (A). Following challenge with 1, 5, 10, or 20 LD_100_ alpha-toxin, three mice per toxin dose were randomly selected for body weight monitoring (B). Similarly, after challenge with C. perfringens at doses of 5 × 10^8^ or 5 × 10^9^, three mice per dose were randomly selected for body weight monitoring (C). Body weight change (%) was defined as the percentage of post-treatment body weight relative to pre-treatment body weight.

**Fig. S5**


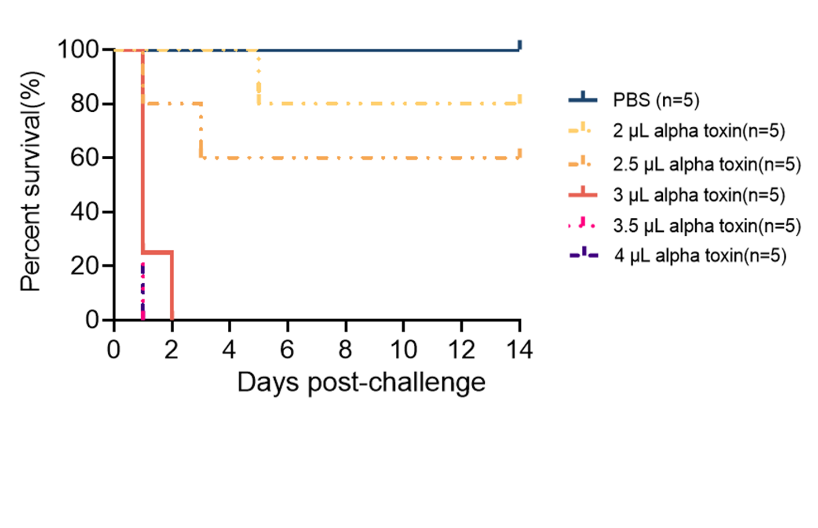


**Fig. S5 Determination of the LD_100_ of crude alpha-toxin in mice.**

The crude α‑toxin used in this study was obtained from *Clostridium perfringens* strain HLJ‑A5, which was genotypically confirmed as a type A strain by PCR, with the following toxin gene profile: cpa (+), cpb (−), etx (−), iap (−), and cpe (−). For α‑toxin production, the strain was cultured in a sterile medium containing proteose peptone, yeast extract, tryptone, ferric sulfate, L‑lysine, and zinc sulfate (pH 7.2). Fermentation was carried out at 35°C for 5 hours. The culture was centrifuged at 10,000 × g for 60 minutes, and the supernatant was passed through a 0.2-µm Pall Supor® DCF filter. The filtrate was concentrated using a Millipore pellicon cassette system containing a high-volume ultrafilter. The toxin was then passed through a sterile Millipore filtration unit containing a 0.22-µm membrane. The preparation process for alpha‑toxin followed the USDA standard protocol for *Clostridium perfringens* Type A (alpha) toxin preparation guidelines (<https://www.aphis.usda.gov/sites/default/files/cvb-dat-0145.pdf>), ensuring reproducibility and consistency with established methods.

To determine the LD100 dose of crude alpha toxin in mice, the crude alpha toxin was diluted with PBS into five concentration gradients, with each 200 µL of PBS containing 2, 2.5, 3, 3.5, or 4 µL of toxin, respectively. Each concentration was administered via intraperitoneal injection to five healthy mice, while a control group (five mice) received an equivalent volume of PBS. After injection, the survival of mice in each group was periodically observed and recorded to plot survival curves. The LD_100_ was defined as the lowest toxin dose resulting in 100% mortality of all injected mice.
